# Comparison of missing data approaches in linkage analysis

**DOI:** 10.1186/1471-2156-4-S1-S44

**Published:** 2003-12-31

**Authors:** Chao Xing, Fredrick R Schumacher, David V Conti, John S Witte

**Affiliations:** 1 Department of Epidemiology and Biostatistics, Case Western Reserve University, Cleveland, Ohio, USA; 2 Department of Preventive Medicine, University of Southern California, Los Angeles, California, USA; 3 Department of Epidemiology and Biostatistics, and Urology, University of California, San Francisco, CA, USA

## Abstract

**Background:**

Observational cohort studies have been little used in linkage analyses due to their general lack of large, disease-specific pedigrees. Nevertheless, the longitudinal nature of such studies makes them potentially valuable for assessing the linkage between genotypes and temporal trends in phenotypes. The repeated phenotype measures in cohort studies (i.e., across time), however, can have extensive missing information. Existing methods for handling missing data in observational studies may decrease efficiency, introduce biases, and give spurious results. The impact of such methods when undertaking linkage analysis of cohort studies is unclear. Therefore, we compare here six methods of imputing missing repeated phenotypes on results from genome-wide linkage analyses of four quantitative traits from the Framingham Heart Study cohort.

**Results:**

We found that simply deleting observations with missing values gave many more nominally statistically significant linkages than the other five approaches. Among the latter, those with similar underlying methodology (i.e., imputation- versus model-based) gave the most consistent results, although some discrepancies remained.

**Conclusion:**

Different methods for addressing missing values in linkage analyses of cohort studies can give substantially diverse results, and must be carefully considered to protect against biases and spurious findings.

## Background

Prospective cohort studies, or longitudinal studies, are generally regarded as being more definitive than case-control studies because they are not subject to numerous potential biases that may affect case-control studies. In particular, the cohort study design entails enrolling a disease-free population at baseline, assessing their exposures at that and future time points, and then comparing the ultimate occurrence of disease among those exposed versus unexposed [1]. Since exposure is assessed prior to the occurrence of disease, cohort studies are not subject to temporal ambiguity and recall bias.

While widely used in epidemiologic research, cohort studies have been rarely used in linkage studies. The preferred study designs for linkage analysis has been large pedigrees, heavily loaded with affected individuals, or affected sibling pairs. However, the incorporation of family information, and continued recruitment into large cohort studies, such as the Framingham Heart Study, has provided a valuable opportunity to undertake linkage analyses in a population-based cohort study. Such studies will allow for temporal linkage analyses, and provide information about genetic risks directly applicable to the general population.

One potential problem with using repeated measures from cohort studies in linkage analyses is the large potential for missing data. Missing data is common in longitudinal studies, and may result in spurious or weakened results, complicating their interpretation [2]. For example, missing data can arise in cohort studies due to subject attrition at individual follow-up points, or complete withdrawal from the study [3].

The effect of missing data on one's results depends on the process underlying the incomplete data collection. This can be classified as follows: 1) missing completely at random (MCAR), wherein the missingness is independent of the observed and unobserved data; 2) missing at random (MAR), wherein the missingness depends only on the observed data; and 3) not missing at random (MNAR), wherein the missingness is dependent upon the missing values only [4]. The presence of the latter two situations may introduce follow-up bias into a study. MAR is less restrictive than MCAR because the probability of the missing value depends only on the observed data [5].

Methods for handling missing data can be categorized with regard to the following four types of procedures: 1) complete subject; 2) weighting; 3) imputation-based; and 4) model-based [4]. The complete-subject approach – the simplest imputation method – removes all individuals with missing data. If missing data is not random among the exposed and unexposed groups, complete-subject analysis may introduce a bias. In addition, complete-subject analysis may be less efficient than other approaches [6]. In the weighting approach, individuals with and without missing data are grouped on variables recorded for both. The nonrespondents receive a weighting of zero, while the matching respondents are assigned a proportionately inflated weight to compensate for the missing values. The imputation-based procedures estimate and fill in the missing values, commonly using mean- and regression-based values, allowing one to use standard analysis methods on a complete data set. Finally, model-based procedures define a model for the missing data and make inferences on the likelihood or posterior distribution under that model [4]. The impact of such methods on linkage analysis of longitudinal data is unclear. Therefore, we investigate here the effect of using six different techniques for handling missing data on linkage analyses in the Genetic Analysis Workshop 13 (GAW13) Framingham data.

## Methods

The sample included 348 pedigrees consisting of 4639 individuals from the Framingham cohort study (GAW13). The population was 49% male and 51% female. The following traits were investigated here: body mass index (BMI); cholesterol (CHL); systolic blood pressure (SBP); and a principal component (PC) variable. The latter was the first principal component of the first three traits (following imputation of their missing values, as described below). As a composite of CHL, BMI, and SBP, the principal component trait attempts to capture the interrelated complexity of these measures in a single trait value. (The proportion of variance attributable to the first principal component ranged from 44% to 56% (mean = 49%).) Trait and covariate information selected from five time points were used within the analysis. The percentage of missing data at the five time points for the variables and covariates is shown in Table 1.

Six different techniques were used to impute missing information for the traits BMI, CHL, and SBP. The first three are imputation-based procedures using a single imputation. Specifically, Method I imputed missing data from the gender-specific mean of the population according to the sex of the individual with missing data. Method II used a linear regression approach: for each individual and each time point, the known values of the trait were used to predict the missing observation. Method III imputed missing data from the gender-specific mean of an individual's pedigree. If the pedigree mean could not be computed due to missing values, then the gender-specific population mean was used instead. The fourth approach (Method IV) was a complete-subject procedure: individuals with missing values were simply removed from the analysis for that (missing) variable. Finally, the last two approaches were model-based single imputations. In particular, Method V modelled the missing values using the expectation-maximization (EM) likelihood-based algorithm. Method VI used the data augmentation Monte Carlo Markov chain method (MCMC) algorithm to model the missing values. Since the traits are quantitative, a Gaussian model was assumed to handle the missing values for both approaches [7]. Both model-based algorithms are implemented in the S-PLUS MISSINGDATA library.

Once missing data were imputed, a univariate linear regression for each of the four traits was performed across time while controlling for smoking (SMK), the number of cigarettes smoked per day, and the number of grams of alcohol consumed per day (ALC). Age served as a proxy for time. Missing age values for individuals were calculated based on the fact that exams occurred every four years for the second generation, except for the first time interval of eight years, and at two-year intervals for the first generation. The family mean for SMK and ALC, adjusted for gender, provided a value for those with missing data for the covariates in the first four methods. In Methods V and VI, the missing covariates and traits were treated identically by using the EM or data augmentation algorithms under a Gaussian model. The linear regression of the four traits provided a coefficient predicting their change, which was then used as the outcome trait in a genome-wide linkage analysis using Haseman-Elston regression [8]. The complete data for Method IV included 1073 observations, while the other five methods had 2885 observations. We used the modified Haseman-Elston method, which computes the mean-corrected cross product as the dependent trait [8]. Seventeen original pedigrees were broken into two separate family pedigrees, one family was broken into four separate family pedigrees, and two pedigrees consisting of 53 individuals were removed in order to assess the identity by descent (IBD) distribution in SAGE [9]. (GENIBD in SAGE breaks pedigrees with more than 18 individuals into nuclear families to compute the IBD distribution.)

## Results

Genome scans with the six imputation methods indicated nominally statistically significant (*p *< 0.05) linkages for the four traits at a total of 107 markers. Method IV gave the largest number of markers with *p *< 0.05 (*n *= 105), while Method III had the fewest (*n *= 19). The number of markers showing *p *< 0.05 for Methods I, II, V, and VI were 38, 32, 23, and 36, respectively. A pair-wise comparison of the significant linkages detected with methods across the three traits, CHL, SBP, and PC, indicated only 3 matches out of 45 (Table 2). However, a pair-wise comparison of the methods with the trait BMI always shows a match. If the methods for handling missing data are of similar type, the matching increases with the BMI trait. In particular, Method I, II, and III agree 72.7-83.4% of the time (I&II: 19/22; I&III: 16/22; II&III: 15/19), while Method V and VI agree 47% of the time. Chromosome 2 has nine significant markers seen only in Method V. Removing this chromosome from the analysis results in the agreement between Methods V and VI increasing to 63%. Method IV agrees with Method I-III, Method V, and Method VI only 1/28, 2/28, and 3/28, respectively. Of the 401 markers, chromosome 10 marker 2 (063 × f4) had significant *p*-values across the six different imputation methods.

## Discussion

Missing data is a critical issue in observational studies, especially those with longitudinal data. Methods for handling missing data have received little attention in genetic epidemiology. Therefore, we undertook an empirical investigation of how six different missing data approaches compared with regard to the number of significant linkages observed across four traits. The six different approaches represent three of the four categories for methods handling missing data: complete subject analysis, imputation-based procedures, and model-based procedures. Since a complete data set was needed for the linkage analysis, the weighting procedure was not performed. Our results demonstrate that there can be substantial variability in linkages when using different methods to handle missing data.

The complete-subject approach (Method IV) generated the most significant *p*-values (*n *= 105), while the other methods gave from 19 to 38 significant *p*-values. Thus, the elimination of individuals with missing values may have introduced a bias into the study, leading spurious results. Most likely individuals with missing values are not MCAR. Those with higher values for the measured health indicators are more likely to miss exams due to poor health or the embarrassment from having previous high values. The smaller sample size used with this method may have also led to an increased number of false-positive results.

The traits we focused on are indicators for health status and are risk factors for many common diseases. For example, CHL, BMI, and SBP are risk factors for cardiovascular disease and stroke. For each trait (CHL, BMI, SBP, and PC), we choose to perform a linkage analysis aimed at identifying the genes involved in the change of these traits through time.

Note that our analyses did not adjust the degrees of freedom for multiple imputation. In practice, this should be done because the imputed values are not truly observed, but instead are based on the existing observed values [10].

Further studies using simulation methods should be conducted comparing the results from different missing data imputation methods. In addition, original data sets from studies finding linkage could be re-examined after using data imputation for comparison and possibly improving previous results.

## Conclusions

Applying different imputation methods for missing data to linkage analyses of longitudinal data can substantially influence one's results. We found that the number of statistically significant results differed quite a bit for each of the six methods used here. As expected, similar types of methods agreed the majority of the time. In summary, while longitudinal studies are critical for evaluating linkage to traits that may vary over time, the treatment of missing data in such studies may greatly affect linkage results and should be considered with caution.

## Figures and Tables

**Table 1:** Percentage of missing values for five time points

	Time7	Time12	Time13	Time14	Time15
BMI	5%	20%	21%	21%	25%
CHL	7%	51%	23%	22%	25%
SBP	5%	20%	20%	21%	24%
ALC	11%	20%	21%	21%	25%
SMK	5%	20%	20%	21%	24%

**Table 2:** Counts of markers with significant *p*-values (<0.05) in four traits comparing six imputation methods in a genome linkage analysis^A^

Method		I			II			III		IV				V		VI
		BMI	SBP	PC	BMI	SBP	PC	BMI	PC	BMI	CHL	SBP	PC	BMI	CHL	BMI
I	BMI	22	0	3	19	0	2	16	0	1	0	2	1	8	1	2
	SBP		1	0	0	1	0	0	0	0	0	0	0	0	0	0
	PC			15	2	0	9	0	1	0	0	0	0	4	0	5
II	BMI				19	0	2	15	0	1	0	2	1	8	1	9
	SBP					1	0	0	0	0	0	0	0	0	0	0
	PC						11	4	0	0	0	0	0	3	0	4
III	BMI							18	0	1	1	2	1	6	1	7
	PC								1	0	0	0	0	0	0	0
IV	BMI									28	10	18	26	2	0	3
	CHL										23	11	9	0	0	0
	SBP											26	17	1	0	2
	PC												27	2	0	3
V	BMI													20	1	17
	CHL														3	1
VI	BMI															36

^A^Traits not listed (for specific methods) did not exhibit any statistically significant linkage results.
